# CT-guided needle lung biopsy is possible during apneic oxygenation: a case series

**DOI:** 10.1186/2049-6958-8-73

**Published:** 2013-12-06

**Authors:** Benedict Kjaergaard, Peter R Zepernick, Annette Bergmann, Henrik K Jensen, Milka Mladenovic, Bodil S Rasmussen

**Affiliations:** 1 Department of Cardiothoracic Surgery, Centre for Cardiovascular Research, and Biomedical Research Laboratory, Aalborg University Hospital, Aalborg, Denmark; 2 Department of Anaesthesiology and Intensive Care, Centre for Cardiovascular Research, Aalborg University Hospital, Aalborg, Denmark; 3 Department of Radiology, Aalborg University Hospital, Aalborg, Denmark; 4 Department of Respiratory Diseases, Aalborg University Hospital, Aalborg, Denmark; 5 Department of Cardiothoracic Surgery, Aalborg University Hospital, Postbox 365, Hobrovej 18-22, DK-9100 Aalborg, Denmark

## Abstract

**Background:**

It can be difficult to perform CT guided biopsy of small pulmonary nodules especially if the position is behind a costa or close to the diaphragm and respiratory movements may hamper the procedure. During apneic oxygenation with a pulmonary standstill these movements can be hindered.

**Methods:**

Six patients with decreased lung function and suspicious lung nodules are presented. Under general anesthesia including a muscle relaxant and a cuffed tube in the trachea CT guided biopsy was prepared. Just before the biopsy the ventilation mode was switched to a continuous positive airway pressure of 5–10 cm H_2_O, maintaining 100% oxygen delivery without ventilation. If the position of the lung nodule was inconvenient for biopsy the pressure was increased to up to 17 cm H_2_O to expand the lungs to a better biopsy position. After retrieving the biopsy controlled ventilation was re-established and a finishing control CT-scan was performed. Blood gas analyses were performed with few minutes interval.

**Results:**

All biopsies were diagnostic. All patients survived the procedure with no major complications, but 3 patients developed pneumothorax. The length of apneic oxygenation was median 10 minutes (8–10 minutes). No major changes in vital parameters were observed, and in all patients the peripheral oxygen saturation was 100% throughout the procedure. The arterial oxygen tension rose to very high values and the lowest pH was 7.18.

**Conclusions:**

It is possible to perform lung biopsies in selected patients with decreased lung function during apneic oxygenation in at least 10 minutes in a safe way.

## Background

In lung cancer establishment of an early diagnosis is important, even if the patient is not suitable for treatment with surgical lung resection. In many cases radiotherapy is a valuable method in the treatment of lung cancer, leading to better survival rates and better quality of life [1]. In other cases, treatment with chemotherapy, and in some cases with targeted chemotherapy such as Gefitinib, is beneficial [2,3]. However, before the initiation of treatment, it is mandatory that a diagnosis is established. Even if new treatment is successful from the start with reduction in tumour size, the tumours may undergo transformation during therapy, which can be a good reason for performing a re-biopsy. If the tumours have transformed and acquired resistance to the initial treatment, it is possible to adjust the treatment in time, by means of the result of a re-biopsy [4].

A CT- guided needle biopsy is a valuable diagnostic tool for the diagnosis of pulmonary nodules and has only few serious complications, except for pneumothorax, which is seen in 1/3 of the patients undergoing this procedure [5]. Although small tumours have the best prognoses, these are more difficult targets to hit with a CT-guided transthoracic needle biopsy, the sensitivity of the procedure thereby being lower. The position of a suspicious pulmonary nodule, e.g. behind a rib, or close to the diaphragm can make it difficult to perform a biopsy, and respiratory movements may hamper it further.

For this, and similar procedures demanding a pulmonary standstill, several alternatives to breathing have been suggested over the years, including passive oxygenation via a catheter inserted into the lungs [6]. However, the accumulation of carbon dioxide and lung collapse have been limiting factors. A well-known method for ventilation of the apneic lungs is jet ventilation [7]. This may also be an option when performing a biopsy of hard-to-reach tissue, however, during jet ventilation you cannot change position of a nodule by expanding the lungs.

Studies of both humans and animals have shown that it is possible to stop ventilation for a prolonged period of time without oxygenation problems, if the lungs are subjected to a constant supply of oxygen, and a continuous positive airway pressure (CPAP) between 5 and 20 cm H_2_O, to keep the lungs open to avoid shunting of deoxygenated blood through the lungs [8-11]. This will lead to a carbon dioxide accumulation, and a corresponding fall in pH to about 7.0 after 30 minutes. It is very important to use 100% pure oxygen, since nitrogen in the gas supply will lead to alveolar nitrogen accumulation with a sudden decrease in blood oxygen saturation [12-14]. This technique is called apneic oxygenation.

In this study we present our first experiences with apneic oxygenation in patients where normal CT-guided needle lung biopsies failed for different reasons.

## Methods and materials

The local Institutional Review Board was consulted and the methods were approved for such pilot study aimed to evaluate if CT-guided lung biopsy is possible during apneic oxygenation. Hereby we present our first 6 consecutive patients. The median age of the patients was 62 years (50–77 years), all patients had decreased lung function with a FEV_1_ median of 44% (32–63%). For various reasons none of the patients were candidates for lung surgery. Three of the patients had previously suffered from cancer. All patients went through our standard diagnostic procedures for patients with suspicious symptoms of lung cancer, including a PET-CT scan, bronchoscopy, endobronchial ultrasound-guided mediastinal lymph node biopsy. The Department of Anaesthesiology and Intensive Care, Aalborg University Hospital, performs approximately 300 CT-guided needle lung biopsies per year, and the 6 patients included in our study have been referred to lung biopsies and included in our study within 1 year, they represent 2% of the approximately 300 patients, that are referred to diagnostic lung biopsies per year.

During the previous attempts to perform lung biopsies, three out of five patients developed a substantial pneumothorax, and were treated with a chest tube. Patients’ characteristics are shown in Table 1.

**Table 1:** Patients’ characteristics

	**Age**	**FEV**_**1**_	**Nodule size**	**Location of nodule**	**CPAP during apnea**	**Pneumothorax after biopsy**	**Diagnosis of biopsy**
Patient 1	69 years	35%	20 mm	Segment 3	17 cm H_2_O	Yes	Malign
Patient 2	50 years	32%	10 mm	Segment 4	10 cm H_2_O	Yes	Malign
				Very central			
Patient 3	77 years	34%	24 mm	Segment 6	10 cm H_2_O	No	Malign
				Pleura near			
Patient 4	77 years	57%	25 mm	Segment 8	6 cm H_2_O	Yes	Malign
Patient 5	52 years	63%	25 mm	Segment 9	8 cmH_2_O	No	Benign
				Diaphragm near			
Patient 6	54 years	53%	10 mm	Segment 10	10 cm H_2_O	No	Malign
				Pleura near			

### The reason for choosing apneic biopsy

Patient 1: This patient developed pneumothorax during the procedure, and was in a critical condition until a chest tube was inserted. Later we realized that the biopsy was inconclusive.

Patient 2: This patient had a very low oxygen diffusion coefficient of 15%, and an attenuated lung structure. To minimize the risk of getting an inconclusive lung biopsy the patient was a preselected candidate to an apneic biopsy.

Patient 3: This patient’s biopsy from a nodule behind a rib turned out to be inconclusive.

Patient 4: This patient’s biopsy turned out to be inconclusive, and because of dyspnoea the first attempt was abandoned.

Patient 5: This patient’s biopsy turned out to be benign, however on a CT scan it appeared very suspicious and because of the nodules’ location close to the diaphragm, it was difficult to achieve a representative biopsy, and therefore it was repeated during apneic oxygenation.

Patient 6: This patient experienced severe coughing and bleeding from the airways during the first unsuccessful attempt to perform a biopsy.

### Anaesthesiology procedure

Prior to lung biopsies in apneic oxygenation all patients were anaesthetized with continuous infusion of propofol (2.3-4.9 mg * kg^-1^ * hour^-1^) and remifentanil (0.23-0.49 μg * kg^-1^* hour^-1^).

A cuffed endotracheal tube was inserted (Potex, Smiths Medical, Watford, United Kingdom) and connected to a Dräger Primus® ventilator (Dräger Medical GmbH, Lübeck, Germany). Normoventilation or slight hyperventilation was obtained using a volume controlled ventilation mode with tidal volumes 6–8 mL/kg, respiratory frequency 12-14/minute, and positive end expiratory pressure (PEEP), 5 cm H_2_O.

Arterial blood samples were drawn for analysis (ABL 800, Radiometer, Copenhagen, Denmark) at baseline, within 1 minute after start of apneic oxygenation and every 5 to 10 minutes during, and after the procedure, until the values were normalized. The patient was placed in the CT scanner and after correct positioning and preparation for biopsy, rucoronium 0.6 mg* kg^-1^ (a muscle relaxing medication) was given to avoid any movements due to carbon dioxide accumulation. A train-of-four (TOF) monitor (TOF-watch®, Organon Ltd, Dublin Ireland) was used to monitor the neuromuscular block. After 2 minutes of pre-oxygenation with 100% oxygen, the ventilation mode was switched to a continuous positive airway pressure (CPAP) of 5–10 cm H_2_O, maintaining 100% oxygen delivery without ventilation. The patient was continuously observed with pulse oxymetry (Dräger Medical GmbH, Lübeck, Germany). If the position of a lung nodule precluded a biopsy, the pressure was increased according to our schedule, not higher than 20 cm H_2_O. The highest value in this small study was 17 cm H_2_O, which expanded the lung to a much better biopsy position. After retrieving the biopsy, controlled ventilation was re-established and a concluding CT-scan was performed to reveal a possible pneumothorax. In case of a clinical symptomatic pneumothorax a chest tube Charrière 18 was inserted. Ventilation was continued until the patient was at his/her own conditions and was normoventilated, or had a satisfactory elimination of carbon dioxide. The patient was transferred to the recovery room and after additional 2–3 hours to the medical ward.

The procedure could only be performed in our largest CT rooms, which normally are used for trauma patients, since we needed space for a bed, a ventilator, equipment for pleural drainage and all technical installations for the ventilator and the monitors.

### Radiologic procedure

The CT-guided needle lung biopsies were performed by an experienced radiologist. CT scanners of various types were used, e.g. GE Lightspeed® Pro 32 slices, GE Lightspeed® VCT and GE Discovery 750 HD both 64 slices (GE, Milwaukee, USA) and Siemens Somatom 10 (Siemens Medical Systems, Erlangen, Germany). The patient was anaesthetized and positioned in the CT scanner. A CT scout view in 2 plans was performed by a preliminary CT scan with 3 mm axial slices of the lung section, containing the lesion of interest. The access point was planned and the distance to the target was measured taking into consideration the topography of the lesion in relation to the surrounding structures. The entry site on the skin was marked with a pen guided by the laser lightning of the CT scanner and followed by antiseptic procedure. No local anaesthesia was used. A small incision was made in the skin, and a 19G coaxial biopsy needle (BARD Truguide Coaxial Biopsy Needle) was placed in the thoracic wall nearby the pleura while checking the progress of the needle by means of small CT scans, 11–13 slices in a CT-biopsy mode. The patient was switched from ventilation to apneic oxygenation and the coaxial needle was placed in the periphery of the lesion, again checking the progress of the needle by means of CT scans. 3 core biopsies were achieved with a biopsy needle 20 G (BARD Monopty Core Biopsy Instrument), and were immediately placed on paper and put in containers with formalin. After that a fine needle aspiration biopsy was performed with a 22 G needle (“Franseen” Biopsy Needle, Angiotech) attached to a 20 ml syringe placed in a handle. Aspirates were smeared on glass slides. Controlled ventilation was re-established.

### Statistics

All values are medians with ranges.

## Results

**Figure 1:**
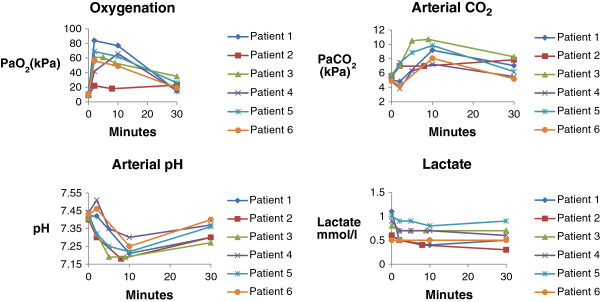
Arterial blood examination during the apneic and recovery period.

All biopsies were diagnostic. The biopsies revealed that 5 out of 6 patients had malignant diagnoses, and the last patient had sarcoidosis. All 6 patients survived the procedure with no major complications, however 3 patients developed a clinical symptomatic pneumothorax and needed pleural drainage for median 1.5 days (1–9 days). In this small study it was not possible to identify any connection between the CPAP values during apnea and pneumothorax. The length of apneic oxygenation was median 10 minutes (8–10 minutes). During the apneic period no major changes in blood pressure or pulse rate were observed, and in all 6 patients the peripheral oxygen saturation was 100% throughout the whole procedure. The arterial oxygen tension rose to very high values, maybe also due to pre-oxygenation and was 55,4 (22,3-83,6) kPa within the first minutes after start of the apneic oxygenation, and at the end of the apneic period the values were 57,3 (18,1-77) kPa. None of the patients experienced a serious drop in blood pH due to carbon dioxide accumulation (Figure 1).

Appropriate treatment for the underlying diseases was instituted in all patients afterwards.

The whole procedure, from initiation of anaesthetization of the patients in the CT room, to end of the procedures in the CT-scan, lasted a relatively long time, approximately 1 ½ hour per patient.

## Discussion and conclusions

In our opinion, apneic oxygenation is an option for improving the accuracy of lung biopsies, in particular in patients with small nodules, and in nodules with positions hard to reach, despite the fact that both fluoroscopy and a CT-guided biopsy mode are performed by an experienced radiologist. By changing the airway pressure to a level between 6 and 17 cm H_2_O, it was even possible to change position of a nodule to facilitate the procedure of biopsy. In this small study it was not possible to investigate the effect of CPAP on pneumothorax. Of course, the procedure demands general anaesthesia and mechanical ventilation for some minutes afterwards to normalize carbon dioxide level, but we still find this procedure more favourable than repetitive biopsy procedures, or even an open surgical procedure to establish the correct diagnosis. We also consider the procedure safe as long as the patient is tightly monitored. The apneic oxygenation can very easily be switched to controlled ventilation if indicated. Even though the patient accumulates carbon dioxide and develops acidosis during apneic oxygenation, this is not as dangerous, as in the critical ill lung patient, since arterial oxygen tension is very high, and since hypercapnia will be reverted immediately after the procedure. We do not know how much pre-oxygenation contributed to the very high values of PaO_2_ measured already within the first minute of apnea, but the values did not fall during the apneic period. However, there are some clear contraindications to hypercapnia such as sickle cell disease, increased intracranial pressure and pulmonary hypertension. We do not know how much the risk of pneumothorax is altered during the procedure. During apnea the slightly elevated airway pressure is at least partly transmitted to the pleural cavity reducing the pressure difference compared to the situation in a patient treated with a ventilator. But when the apneic oxygenation is switched to the ventilator, pneumothorax may develop and a preparedness for chest drainage is necessary, especially because tension pneumothorax is a risk after resumption of mechanical ventilation. It is unclear if apneic oxygenation may increase the risk of air embolism, but the airway pressure during apnea is not as high as the peak pressure during coughing. Air embolism happened in less than 1‰ of conventional lung biopsies in 2 big series that included more than 15,000 patients [5,15].

Optimizing the biopsy procedure includes the participation of a pathologist, who performs immediate preliminary microscopy, insuring that the biopsy is representative. The time spent in the CT room could be reduced, if we had a room for inducing anaesthesiology adjacent to the CT room at our disposal. There may also be possibilities in stereotactic devices for CT-guided lung biopsy when the patient does not move during the procedure.

Treatment with apneic oxygenation may even be useable for stereotactic radiotherapy and for radiofrequency ablation for lung cancer.

## Consent

All patients included gave informed consent to participation to the study.

## Competing interests

The authors have no commercial association or financial involvement that might pose a conflict of interest in connection with this article. There has been no financial support to this work.
